# Nanog Variability and Pluripotency Regulation of Embryonic Stem Cells - Insights from a Mathematical Model Analysis

**DOI:** 10.1371/journal.pone.0011238

**Published:** 2010-06-21

**Authors:** Ingmar Glauche, Maria Herberg, Ingo Roeder

**Affiliations:** 1 Institute for Medical Informatics, Statistics and Epidemiology, Medical Faculty, University of Leipzig, Leipzig, Germany; 2 Institute for Medical Informatics and Biometry, Faculty of Medicine “Carl Gustav Carus”, Dresden University of Technology, Dresden, Germany; Fondazione Telethon, Italy

## Abstract

The expression of the transcription factors Oct4, Sox2, and Nanog is commonly associated with pluripotency of mouse embryonic stem (ES) cells. However, recent observations suggest that ES cell populations are heterogeneous with respect to the expression of Nanog and that individual ES cells reversibly change their Nanog expression level. Furthermore, it has been shown that cells exhibiting a low Nanog level are more likely to undergo differentiation. Applying a novel mathematical transcription factor network model we explore mechanisms and feedback regulations to describe the observed variation of the Nanog levels in mouse ES cells. In particular we show that these variations can occur under different assumptions yielding similar experimental characteristics. Based on model predictions we propose experimental strategies to distinguish between these explanations. Concluding from our results we argue that the heterogeneity with respect to the Nanog concentrations is most likely a functional element to control the differentiation propensity of an ES cell population. Furthermore, we provide a conceptual framework that consistently explains Nanog variability and a potential “gate-keeper” function of Nanog expression with respect to the control of ES cell differentiation.

## Introduction

Embryonic stem (ES) cells are derived from the inner cell mass of the blastocyst. Under appropriate culture conditions these cells can be maintained and expanded in an undifferentiated state over many passages, a feature commonly referred to as *self-renewal*. Furthermore, ES cells are *pluripotent* in the sense that they have the ability to differentiate in a multitude of different cell types in vitro and to contribute to chimera formation after reinjection into a blastocyst [1], [2], [3], [4].

The maintenance of the pluripotent state of ES cells over many self-renewing divisions is associated with a characteristic (and stabilized) expression pattern of particular genes. Over the last years it has been demonstrated that especially the transcription factors Oct4 and Sox2 play a crucial role in this maintenance process by directing the gene expression in ES cells through a cooperative interaction [5]. Knockout experiments of either Oct4 or Sox2 disabled the self-renewal ability of ES cells [6], [7]. This duo of transcription factors (TF) is supported by a third factor, Nanog, which seems to be almost equally important for self-renewal. Early studies on the function of Nanog revealed that the absence of Nanog leads to cell differentiation and the loss of pluripotency both in vivo and in vitro. It was demonstrated that Nanog-null embryos do not develop beyond implantation and that the RNAi-mediated knock-down of Nanog induces the differentiation of ES cells along specific lineages [8], [9]. Conversely, it has been shown that targeted overexpression of Nanog obviates the requirement for extrinsic signals to block differentiation, therefore, preventing ES cells from differentiation under conditions in which they would otherwise differentiate [10].

Although the importance of the three factors Oct4, Sox2 and Nanog for the regulation of pluripotency and differentiation is well established, their mutual interaction and the resulting regulatory dynamics are only incompletely understood. It is known that Oct4 and Sox2 form heterodimers which regulate several ES cell specific genes, including Oct4 and Sox2 themselves as well as Nanog [7]. However, the role of Nanog in this interplay is still controversially discussed. New experimental results provide evidence that the maintenance of pluripotency does not necessarily require continuously high Nanog levels. Instead, it could be shown that Nanog levels are variable and reversibly changing under appropriate self-renewal conditions [11]. Although cells with high Oct4 and Sox2 but low Nanog levels can in general be kept in an undifferentiated, pluripotent state, it has also been demonstrated that these cells are more prone to differentiation as compared to cells that express high levels of Nanog [11]. These findings support the hypothesis that the Nanog-low state is a transient and reversible state that acts as a temporally restricted “gate-keeper” for extrinsic signals by which ES cells are directed towards differentiation [12], [13]. Therefore, the molecular control of Nanog levels has to be regarded as an important piece in the puzzle of pluripotency organization as it is a potential candidate mechanism to maintain the balance between self-renewal and differentiation. A detailed quantitative understanding of these processes and their consequences on the system dynamics would also be highly beneficial for the development and the optimization of reprogramming protocols including the generation of induced pluripotent stem (iPS) cells from somatic stem cells or even fully differentiated cells [14], [15], [16], [17], [18]. Recent analyses have shown that Nanog is required in order to facilitate the final stages of somatic cell reprogramming to a pristine pluripotent state [19].

The experimental observation of reversibly changing Nanog levels in pluripotent ES cells raises the question whether this variation is a functional feature of the regulation network or whether it is just a random fluctuation without regulatory consequences. As the dynamics of (even simple) regulatory networks are hardly predictable based on biological intuition alone, a systems biological analysis, i.e. the application of mathematical modelling techniques, appears to be an appropriate strategy to address the principles of regulatory interactions in pluripotent cells.

In this paper we analyse the dynamics of an interaction network between the transcription factors Oct4, Sox2 and Nanog with a particular focus on potential mechanisms of the observed Nanog fluctuations. The proposed model is based on a set of simple and experimentally motivated assumptions and can be considered as “minimal” (i.e. most simple) in the sense that it concentrates on basic regulatory processes intentionally neglecting the influence of secondary effectors. In particular, we present two fundamentally different mechanisms that can explicitly account for variations of the Nanog levels in ES cells and analyse them using a set of coupled ordinary and stochastic differential equations. Furthermore, we outline a set of experimental approaches to test and to distinguish between these mechanisms. In contrast to other model approaches [20], [21], [22] we relax the assumption that the relevant TF are coupled by strictly positive feedback loops as it is evident that within such a setup, variations of Nanog ultimately and directly entail variations in the levels of Oct4 and Sox2. Such a synchronized variation of all three TF contradicts present experimental findings and, therefore, shows the necessity of a new model description. Building upon the proposed core network of the pluripotency genes Oct4, Sox2 and Nanog we present and discuss a novel concept explaining the suggested “gate keeper” effect of Nanog and demonstrate its consistency with experimentally observed phenomena.

## Methods

### Conceptual framework

**Figure 1:**
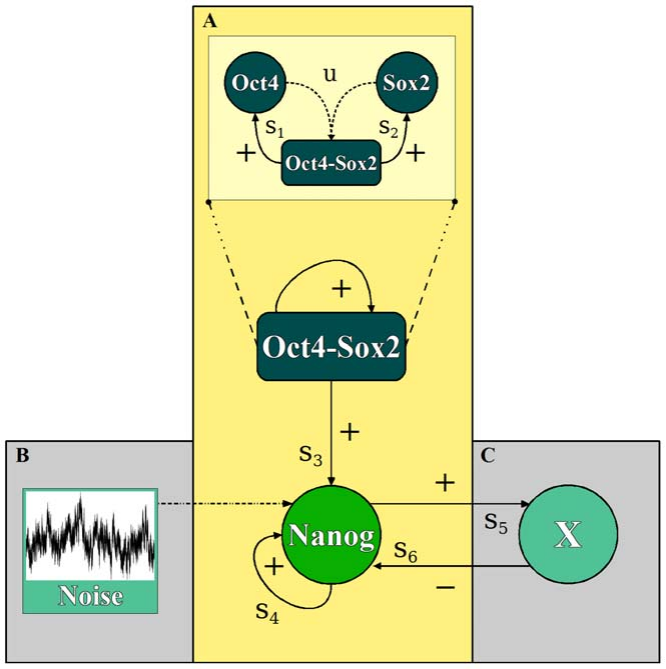
Conceptual scheme of the assumed interaction network. (**A**) Core network for the interaction between Oct4-Sox2 heterodimer and Nanog: positive auto-regulation of the Oct4-Sox2 complex (with complex formation/transcription rates u, s_1_, s_2_, see inset), transcription activation of Nanog by Oct4-Sox2 (with rate s_3_) and auto-regulation of Nanog (with rate s_4_). The inset illustrates the formation of the Oct4-Sox2 heterodimer (with rate u) which in turn activates transcription of Oct4 and Sox2 (with rates s_1_ and s_2_, respectively). (**B**)**,**(**C**) represent the additional regulatory components which complement the core network: (**B**) stochastic fluctuations acting on the Nanog transcription and (**C**) the additional factor X establishing a negative feedback on Nanog (with rates s_5_ and s_6_), thus generating an activator/repressor system.

The interaction dynamics between the TF Oct4, Sox2 and Nanog are described in terms of their intracellular protein concentrations. The temporal changes of these concentrations are represented by a set of coupled ordinary differential equations. Briefly summarizing the relevant experimental observations, we motivate a set of minimal assumptions that form the basis of our modelling:

(1) There is evidence that the regulation of Oct4 and Sox2 is primarily facilitated by the action of an Oct4-Sox2 heterodimer [7]. This complex maintains the constantly high Oct4 and Sox2 expression through a positive and self-reinforcing regulatory loop in pluripotent cells (Figure 1, frame (A)). In ES cells it also acts on other target genes such as Nanog, FGF4, Utf1 and Fbx15 [5], [7]. Assuming that the concentrations of the Oct4-Sox2 heterodimer is always in a dynamic equilibrium with the concentrations of the Oct4 and Sox2 proteins, and that the heterodimer is the major regulatory complex for the transcriptional regulation, it is a reasonable simplification to solely account for the concentrations of the heterodimer, instead of the single proteins. Therefore, within our model description the formation of the Oct4-Sox2 heterodimer and its transcriptional regulation of Oct4 and Sox2 expression are replaced by an auto-regulation of the heterodimer (Figure 1, inset in frame (A)). The temporal dynamics of the Oct4-Sox2 heterodimer are, in this case, determined by a positive auto-regulation of the heterodimer itself and an exponential decay as the result of assumed first-order degradation kinetics of this complex.

(2) The dynamic behaviour of the Nanog concentration is described in terms of a positive Nanog auto-regulation [23] mediated by Nanog dimer molecules [24], [25], an additional activation that depends on the concentrations of the Oct4-Sox2 complex [5] as well as an exponential decay of Nanog (i.e. assuming first-order degradation kinetics). In the unperturbed situation the Oct4/Sox2 auto-regulation results in a stable concentration of the Oct4-Sox2 heterodimer which translates into a (constant) basal transcription for the downstream Nanog.

The combination of assumptions (1) and (2) results in a simple core interaction network that is described in terms of the Oct4-Sox2 heterodimer and the Nanog concentrations (Figure 1, frame (A)). For reasons of simplicity, interactions between the TF are solely described on the transcriptional level. That means, we intentionally neglect other (e.g. post-transcriptional) regulatory effects and argue that the relevant protein concentrations linearly correspond to the mRNA concentrations right after transcription.

Technically, we start with a set of ordinary differential equations for the Oct4/Sox2 and Nanog concentrations which are derived on the basis of stoichiometric chemical equations describing the transcription factor binding to gene promoters. The activation and the repression of transcription factors are represented by S-shaped functions that describe the transcriptional activity as a function of the concentration of the activator or repressor (i.e. *Hill functions*). Furthermore, we assume that all transcription factors decay with first order kinetics. For the Nanog expression two major cis-regulatory regions have been uncovered, a distal enhancer and a proximal promoter. The enhancer is reported to be occupied and positively regulated by several factors such as Klf4, STAT3 and a Nanog-Sall4 complex, whereas the Oct4-Sox2 complex is shown to regulate the proximal promoter [26]. Therefore, we assume for the transcriptional regulation of Nanog that the activation by the Oct4-Sox2 complex and Nanog itself are independent from each other, thus resulting in an additive activation term.

The equation for the concentration of the Oct4-Sox2 heterodimers ([OS], Eq. 1) and for the concentration of Nanog ([N], Eq. 2) within the core network (Figure 1, frame (A)) are given by 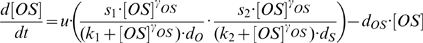
(1)

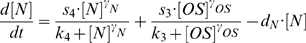
(2)


The formation of the Oct4-Sox2 heterodimer occurs at rate u. The parameters s_i_ denote the transcription/repression rates, k_i_ the equilibrium (dissociation) constants, and the coefficients γ_OS_, and γ_N_ determine the shape of the response function (generally termed *Hill coefficients*). The degradation rates of Oct4, Sox2, Nanog and of the Oct4-Sox2 complex are denoted by d_O_, d_S_, d_N_ and d_OS_ respectively.

For the given ODE system there exists a parameter region in which stable Nanog concentrations can be observed at either high or low levels. More technically speaking, two stable fixed points exist simultaneously and the system can be trapped in either of the two. Such behaviour is generally referred to as *bistability*. However, the experimentally observed variations of the Nanog levels within individual ES cells show that the regulation of Nanog levels is a dynamic phenomenon (i.e. the cells change reversibly between the high and the low state). This, however, cannot be explained by the existence of two stable fixed points alone. Instead, an additional dynamical process that enables the switch between the stationary system states is required. In principle, there are different possible scenarios to account for such a behaviour. To investigate the general principles of the regulatory effect of varying Nanog levels, we will restrict ourselves to two qualitatively different scenarios. These are (I) stochastic, noise-induced transitions between the Nanog high and the Nanog low state (Figure 1, frame (B)) and (II) an oscillatory pattern due to the negative feedback of an additional regulatory component, termed X (Figure 1, frame (C)). The necessary assumptions are outlined below:

### (I) Stochastic fluctuations of the Nanog expression

To account for a potential stochastic component in the regulatory dynamics we assume that TF expression is at least partially a noisy process (Figure 1, frame (B)). That means, TF expression contains a stochastic part, which is described in the model by a Gaussian white noise term. The assumption of a Gaussian White Noise represents an approximation of multiple heterogeneous sources of noise that might occur on the molecular level. As we here focus on the analysis of general principles of molecular heterogeneity, but not on an explicit modelling of the mechanisms generating the noise, the White Noise approximation is considered appropriate for the scope of the presented models. For this *fluctuation scenario* the temporal changes of the Nanog concentration are given by a stochastic differential equation of the form:
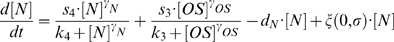
(3)


Herein the ODE (Eq. 2) is complemented by a stochastic term ξ implemented as zero-mean 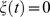) Gaussian process in which σ defines the noise amplitude. Negative Nanog concentrations are excluded by setting [N] = 0 if the concentrations fall below zero. This technical simplification is reasonable for small to moderate values of the noise amplitude σ.

This stochastic part, which can be interpreted as a background transcription, is added for two reasons: First, it has been shown that cellular properties, especially in stem cell populations, show a certain level of inherent heterogeneity [27], [28] for which the stochastic background transcription is a suitable representation. Second, there is evidence, that stochastic fluctuations of gene expression levels potentially have a functional role and contribute to cellular stability. They might even be involved in the control of cell fate decisions [29], [30]. The impact of this assumption is discussed below.

### (II) Oscillations of the Nanog expression

In order to study the possibility of an additional oscillatory behaviour of the transcription factor network, we hypothesize about extending the proposed core network by at least one negative feedback loop [31]. Motivated by the repressive action of transcription factors such as p53, Tcf3 or the NODE complex (Nanog and Oct4 associated deacetylase) [32], [33], [34], we add another regulatory component termed X (e.g. another TF or TF complex) to the core network, which is assumed to be positively regulated by Nanog and in turn negatively regulates the transcription of Nanog (Figure 1, frame (C)).

For this scenario (referred to as *oscillation scenario*) the system of ODEs is extended by a transcriptional repressor X occupying binding sites at the Nanog promoter. Therefore, Eq. (2) is amended to include the competitive repression and reads 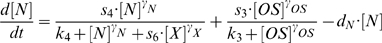
(4)


The repressive action of X could also target the Oct4/Sox2 promoter. However, this leads to qualitatively similar results and is therefore not further considered.

The respective equation for the repressor X is given as
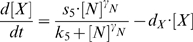
(5)


As above, the parameter d_X_ describes the degradation of X while γ_X_ corresponds to the Hill coefficient of the corresponding response function.

The stated ODE system in Eqs. (1), (4) and (5) is used to analyse the general system properties such as the existence, the stability, and the parameter dependence of stationary states. To model the effects of the assumed stochastic background transcription and to simulate individual trajectories of the Nanog expression in individual cells as well as of Nanog distributions within populations of cells, we will again complement the ODE description of the Nanog dynamics with a white noise term, resulting in the following stochastic differential equation (SDE, replacing Eq. (4)): 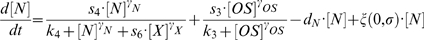
(6)


### Model parameters

The detailed dynamics for each scenario depend on the choice of the model parameters. In both scenarios the noise strength σ and the particular rate constants are adjusted in a way that (a) the fraction of Nanog-low cells within an ES cell population is about 20% and that (b) the concentrations in the different Nanog states differ from each other by two orders of magnitude as observed experimentally [11], [35]. The default parameter sets used for simulations of the fluctuation and the oscillation scenario are given in Table 1. If modifications of these reference parameters values have been used these are noted at the respective legend.

**Table 1:** Parameter sets for both scenarios. n/a codes for “not applicable” in the particular scenario.

Parameter	Values of the Fluctuation scenario	Values of the Oscillation scenario	Parameter	Values of the Fluctuation scenario	Values of the Oscillation scenario
u	0.03°	0.03°	f	270	16
s_1_	50*	50*	d_O_	1#	1#
s_2_	50*	50*	d_S_	1#	1#
s_3_	0.1*	0.1*	d_OS_	1#	1#
s_4_	14*	30*	d_N_	1#	1#
s_5_	n/a	10.08*	d_X_	n/a	0.2#
s_6_	n/a	4.74	γ_OS_	1	1
k_1_	10	10	γ_N_	2	2
k_2_	10	10	γ_X_	n/a	1
k_3_	10	10	σ	12	0.3
k_4_	10	2			
k_5_	n/a	10			

° 1/(molecules × time unit).

*molecules/time unit.

# 1/time unit.

To study the parameter dependence of the system dynamics in the fluctuating scenario we performed bifurcation analysis, using the auto-regulatory transcription rate s_4_ as bifurcation parameter. This parameter has been chosen as it represents the major regulator of the Nanog concentration in the model. In the oscillation scenario the ratio between the transcription rate s_5_ and the repression rate s_6_ is decisive for the oscillation pattern. Therefore, s_6_ is used as bifurcation parameter.

For the quantitative comparison of the scenarios we introduced a normalization factor f such that the mean residence time of cells in the Nanog-low state is normalized to 1 in both scenarios. This results in a normalized time scale τ = f • t with arbitrary units that is used throughout this publication. A rescaling of the time axis to biologically meaningful units can be achieved once more consolidated experimental data (e.g. on the time to the re-establishment of the bimodal Nanog distribution from purified Nanog high or low subpopulations) are available.

### Simulation procedure

The stability and the bifurcation analyses have been realized using the software tool *xppaut* (www.math.pitt.edu/~bard/xpp/xpp.html). The Euler-Maruyama method has been applied to approximate numerical solutions of the stochastic differential equations. The simulations have been implemented using the programming language Perl (www.perl.org).

## Results

### Nanog fluctuations as the consequence of stochastic state transitions (“fluctuation scenario”)

**Figure 2:**
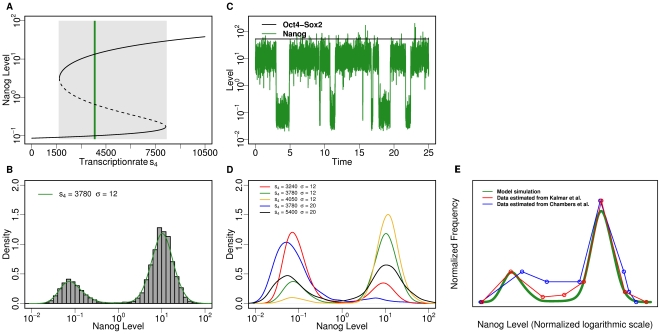
Simulation results for the *fluctuations scenario*. (**A**) Bifurcation diagram, showing the stable (solid black line) fixed points (FP) of the Nanog concentration as a function of the auto-regulation parameter s_4_. Unstable FP are indicated by the dashed line. The light grey area indicates the region of bistability. (**B**) Distribution of Nanog levels within a cell population (5000 cells with identical parameters s_4_ = 14 and σ = 12). The green curve estimates the density function. (**C**) Typical time course (trajectory) of Nanog (green) and Oct4-Sox2 (black) concentrations (parameter choice: s_4_ = 14 and σ = 12). (**D**) Varying interaction parameters (s_4_ and σ) induce changes in the shape of the bimodal distribution indicated by the density functions. (**E**) Comparison of the simulated, bimodal distribution of Nanog levels (green, identical to (B)) with corresponding flow cytometry data estimated from Kalmar et al. [35] (red) and Chambers et al. [11] (blue). The curves are normalized and shifted to match the local maxima for high Nanog levels.

The existence of a positive feedback loop is a necessary prerequisite to generate multistationarity [36]. The positive regulation of Nanog by its own dimer [24] fulfils this criterion and is sufficient for the existence of a bistable regime, i.e. the theoretical possibility to generate a system in which Nanog is stably expressed in either of two different states. In our particular case this means that for certain parameter values of the transcription rates s_1_ to s_4_ the Nanog concentration stabilizes either at a low level, i.e. close to a basal transcription rate mediated by the Oct4-Sox2 complex, or at a high level with substantial contribution from the auto-regulation. These stable expression levels are referred to as stable stationary states or fixed points of the system. The solid lines in the bifurcation diagram (Figure 2A) illustrate these *stable* fixed points of the Nanog concentration as a function of the auto-regulative transcription rate s_4_ (chosen as bifurcation parameter). Within a certain parameter region with respect to s_4_ two stable fixed points exist simultaneously (*bistability*, grey shaded in Figure 2A). An unperturbed system would be trapped in either of the two states. The stable fixed points are separated by an additional *unstable* fixed point, illustrated by the dashed line. As real world systems will always escape from such an unstable state (even due to vanishingly small perturbations), this state is of no practical relevance and will, therefore, be excluded from our discussion.

The introduction of perturbations, either by transcriptional (background) noise, by targeted over-expression, or both, can in principle switch the system from one to the other fixed point and back depending on the magnitude of perturbation. Studying the role of functional noise on the Nanog expression, we identified a parameter region within the bistable regime in which the magnitude of a noisy (background) transcription can be adjusted such that the system reversibly changes between the upper and the lower state of Nanog concentrations. A typical trajectory of the Nanog concentrations within an individual cell in this regime is shown in Figure 2C. The corresponding parameter value s_4_ is given by the green line in Figure 2A.

To compare flow cytometry measurements of Nanog concentrations within a heterogeneous cell population with the results of our model analysis, we simulated the Nanog concentration in a large number of individual cells per time point. Herein each cell represents an independent realization of the outlined, stochastic molecular model. Using such an approach, one observes a bimodal distribution of the Nanog levels (Figure 2B). This behaviour is consistent with experimental findings [11], [35] (Figure 2E). Whereas the left peak represents the cells that currently express low levels of Nanog, the right peak corresponds to the cells with high Nanog concentrations. Changes in the shape of this stationary distribution are shown in Figure 2D for varying values of the transcription rate s_4_ and the variance σ^2^ of the “white noise” term.

The transition probability between the Nanog-low and Nanog-high state depends on the parameter choice with respect to the transcriptional activity (e.g. on the transcription rate s_4_) as well as on the degree of the transcriptional noise (characterized by the variance σ^2^). These transition probabilities, both, for the low-to-high as well as for the high-to-low transition, critically regulate the residence times within each of these states. As we assume the transition probabilities to be constant over time, the distribution of the residence times approaches an exponential distribution (see discussion below).

We point out that this scenario of a noise-induced transition between the two Nanog states does not require the existence of any further feedback loop within the core network to consistently explain the experimentally observed Nanog variations. The effect of an additional negative feedback regulation will be discussed in the next paragraph.

### Nanog oscillations as the consequence of a negative feedback loop (“oscillation scenario”)

Beside the noise-induced state transition within a bistable system, an alternative explanation of Nanog level variations can be provided by oscillatory system dynamics. It has been shown mathematically that at least one negative feedback loop is required to generate oscillatory patterns in systems that do not consider time delays in the regulatory processes [37]. Therefore, we extend the core network of Oct4, Sox2 and Nanog by adding a hypothetical factor X (compare Materials and Methods). The assumed interaction between X and Nanog represent a typical activator/repressor system as the transcription of factor X is assumed to be activated by Nanog but X conversely represses Nanog transcription at the same time (Figure 1, frame (C)). Experimental strategies to characterize potential candidates for such a cofactor are discussed below.

**Figure 3:**
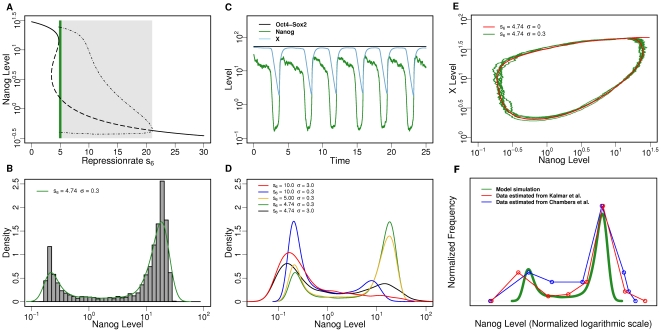
Simulation results for the oscillation scenario. (**A**) Bifurcation diagram, showing the Nanog level as function of the repression rate s_6_. The light grey area indicates the region where oscillations can be observed. The dash-dotted lines represent minimum/maximum values of the oscillations. Solid lines and dashed lines (outside the grey area) represent stable and unstable fixed points, respectively. (**B**) Distribution of Nanog levels within a cell population (10000 cells with identical parameters s_6_ = 4.74, σ = 0.3). The green curve estimates the density function. (**C**) Typical time course of Nanog (green), Oct4-Sox2 (black) and X (blue) concentration for s_6_ = 4.74, σ = 0.3. (**D**) Varying interaction parameters/noise levels (s_6_, σ) induce changes in the shape of the bimodal distribution as outlined by the density functions. (**E**) The phase space diagram visualizes the cyclic behaviour of the Nanog and the X concentration (red line – without noise, green line – with noise). (**F**) Comparison of the simulated, bimodal distribution of Nanog levels (green, identical to (B)) with corresponding flow cytometry data estimated from Kalmar et al. [35] (red) and Chambers et al. [11] (blue). The curves are normalized and shifted to match the local maxima for high Nanog levels

In contrast to the situation of noise-induced transitions in the bistable setting (i.e. the fluctuation scenario), the oscillatory system is characterized by the existence of a so-called *limit cycle* attractor. This means, for certain parameter values the system asymptotically converges to a behaviour in which the concentrations of Nanog and X change in a predictable, periodic fashion, independent of the initial conditions. The parameter region (with respect to repression rate s_6_, chosen as bifurcation parameter) which accounts for such an oscillatory system behaviour is shown by the grey-shaded region in the bifurcation diagram in Figure 3A. In contrast to the solid black lines outside this shaded area, which again represent stable fixed points, the solid black lines within the shaded region illustrate the minimum and maximum value of Nanog expression between which the system oscillates for a given value of parameter s_6_.

To illustrate the typical system behaviour, Figure 3E shows a phase space diagram which visualizes the concentration of Nanog vs. the concentration of factor X. Herein the attraction to the limit cycle starting from a given initial value is shown in red. However, due to the assumed stochastic component of Nanog transcription, the actually realized oscillations do not show a strictly fixed period, but are shortened or prolonged in a stochastic manner (example trajectories in the Nanog vs. X phase space are shown in green in Figure 3E). A typical time course of the concentrations of Nanog and X within an individual cell, together with the corresponding constant expression level of Oct4/Sox2 is provided in Figure 3C.

Accessing the distribution of Nanog concentrations in a population of many cells, the resulting histogram again shows a bimodal characteristic in which the left peak corresponds to Nanog-low cells and the right peak comprises the Nanog-high states (Figure 3B). A comparison to experimental data is provided in Figure 3F. As the frequency and the shape of the oscillations is governed by the interaction strengths between Nanog and its repressor, which is controlled by the rate constants s_5_ and s_6_, these parameters do also influence the fractions of cells in Nanog-high vs. Nanog-low state. As a consequence, changes in these parameters sensitively affect the shape of the resulting bimodal distribution (Figure 3D). Although also the degree of the stochastic component σ^2^ slightly effects the stationary Nanog distribution, it should clearly be pointed out, that in this oscillatory scenario the changes between the Nanog-high and the Nanog-low state are not induced by the transcriptional noise of Nanog but are the result of the activator/repressor system between Nanog and the co-regulator X. However, the variations in the period of the oscillations of the activator/repressor system are induced by the noise term. This implies, as a further result, that in the absence of a stochastic component (i.e. σ^2^ = 0) the residence times in either the Nanog-high or the Nanog-low state are fixed. Upon induction of the stochastic component (σ^2^ >0) the distributions approach a rather symmetric and clearly peaked shape (see below) which is clearly distinct from the exponential-like distribution in the above case of stochastic state transitions.

### Model predictions and proposed experimental strategy

On the cell population level (i.e. with respect to the established distributions of Nanog levels) the two discussed scenarios are phenomenologically indistinguishable. However, as the nature of the Nanog variation differs in the scenarios, we propose two experimental strategies which are predicted to distinguish between them.

**Figure 4:**
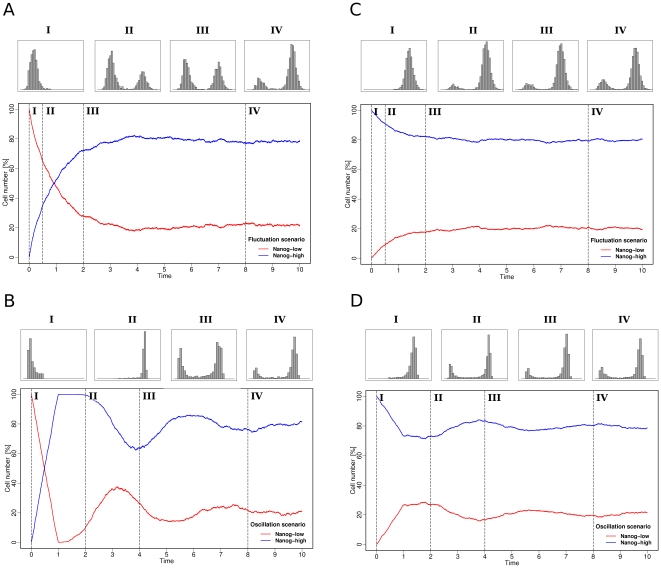
Time courses of the re-establishment of the bimodal distribution. (**A,C**) Fluctuation scenario: starting from the bimodal distribution, 10000 model cells are sorted in Nanog-low cells (red) and Nanog-high cells (blue). Re-establishment of the initial bimodal distribution starting from only Nanog-low (**A**) or Nanog-high (**C**) cells is shown as a function of time (arbitrary time scale). Representative distributions (histograms) of the Nanog concentration within the whole population are shown above for four selected time points. (**B,D**) Corresponding visualization for the oscillation scenario.

The first approach is based on the sorting of a (heterogeneous) population of ES cells into Nanog-high and Nanog-low cells and the subsequent culture of these (purified) subpopulation under self-renewal conditions. Such experiments have already been done, demonstrating in principle that the original Nanog distribution is re-established from both subpopulations on a typical time scale of about 10–13 days [35]. Although such a re-establishment is predicted by our model for both above described scenarios in a similar manner, we argue that the *dynamics* of this process differ. For the first scenario, in which transitions between the Nanog-low and the Nanog-high state are the result of the stochastic fluctuations of the Nanog expression itself, we predict the re-establishment of the bimodal distribution to appear as a continuous shift from the preselected population back into the dynamical equilibrium (Figure 4 A and C). In contrast, for the scenario in which the Nanog variation is induced by the oscillatory behaviour of an activator/repressor system, the sorting of Nanog-low or high cells actually corresponds to a synchronization of the selected cells in two particular (opposing) parts of the limit cycle. In case of selection for Nanog-low cells, most of these cells are rather simultaneously shifted toward the Nanog-high state and then back again. Depending on the magnitude of the assumed background noise (i.e. the size of σ^2^), this synchronization is lost over time and the stabilized equilibrium between Nanog-high and Nanog-low states is re-established. In particular, one observes damped oscillation behaviour in the activator/repressor system (Figure 4 B and D). Caused by the synchronization (which is induced by the sorting procedure), the selected cells are predicted to transiently lead to a higher fraction of cells in the opposing state (“over-shooting”) as compared to the stabilized equilibrium. This “over-shooting” is specifically pronounced in the situation of initially selecting Nanog-low cells (Figure 4 B). A comparable phenomenon is not expected for a bistable system with stochastic transition between the Nanog-low and Nanog-high states, where an aperiodic convergence into the stabilized bimodal distribution can be observed (Figure 4 A and C).

For the detection of a potential system with oscillatory behaviour, the fraction of Nanog-high vs. Nanog-low needs to be determined on a small time scale. As the oscillatory period can only be estimated in the order of a few hours up to a few days, a fine scale of measurements is ultimately required for such an experiment.

The second suggested experimental strategy is based on the continuous monitoring of the cellular development on the single cell level. Such an approach can be facilitated by the application of time lapse video microscopy, given the availability of a molecular (fluorescence) reporter linked to the Nanog protein that allows the determination of the intracellular Nanog concentrations.

**Figure 5:**
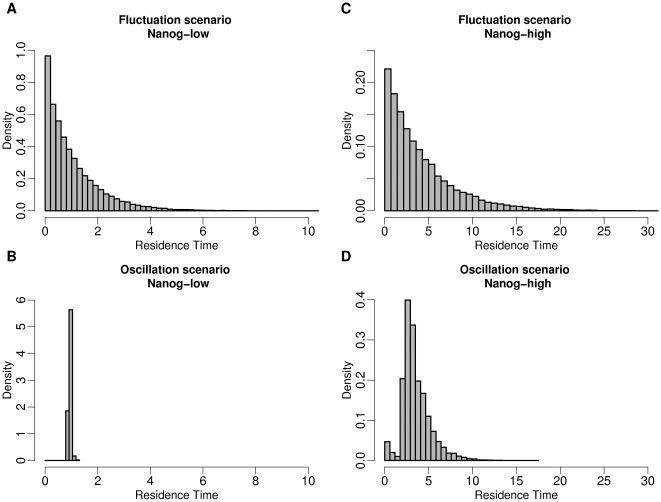
Distribution of residence times. The distributions of the residence time (arbitrary time scale) in the Nanog-low state (**A**) and Nanog-high state (**C**) are shown for the noise-induced fluctuation scenario (s_4_ = 14, σ = 12). (**B,D**) Corresponding distributions of the residence time for the oscillation scenario in the Nanog-low state (**B**) and Nanog-high state (**D**) (s_6_ = 4.74, σ = 0.3).

Using such a system, the overall Nanog expression within an ES cell population should give the same readout as the cell sorting approach illustrated in Figure 4, allowing to detect or to exclude oscillatory behaviour. Moreover, using such a single-cell tracking system it would be possible to directly determine the spatial arrangement and the residence time of individual cells in both the Nanog-high and the Nanog-low state. As stated above, for the case that variation in the Nanog levels result as the consequence of stochastic state transitions (fluctuation scenario), we predict that the distribution of residence times approaches an exponential distribution. This implies that rather long residence times are less likely but still occur with non-zero probability. In contrast, if the Nanog variation is the result of an oscillating activator/repressor system (oscillation scenario), the residence times are expected to show rather symmetric distributions in which the magnitude of the noise term determines the variance. In such a scenario the probability for extreme events is significantly lower. Figure 5 illustrates the distribution of simulated residence times in the Nanog-high and Nanog-low states for both scenarios. In case of the fluctuation scenario, the simulated distributions for the residence times in the Nanog-high as well as the Nanog-low states reflect the predicted exponential distribution (Figure 5A and C). In contrast, the corresponding distributions for the oscillation scenario are clearly peaked around a maximum value which is about the oscillation period of the system without the additional noise term (Figure 5B and D).

### Nanog concentration as the central element to control ES cell differentiation

As stated above, a consistent explanation of reversible Nanog concentration levels together with constant levels of Oct4 and Sox2 expression in pluripotent mouse ES cells does not permit a substantial direct transcriptional regulation of Oct4 and Sox2 by Nanog as assumed by previous modelling approaches [20], [22]. In fact the inclusion of a direct feedback mechanism, as suggested by binding sites analysis [23], contradicts currently available data on fluctuating Nanog levels as the fluctuations would ultimately propagated on the direct target genes. Nevertheless it appears that Nanog concentration is a critical regulating element to control the propensity of ES cells to undergo differentiation [11], [13]: as long as Nanog is present in high concentrations the cell is protected from differentiation inducing signals while in the case of low Nanog levels such signals can initiate differentiation including the ultimate down-regulation of Oct4 and Sox2.

**Figure 6:**
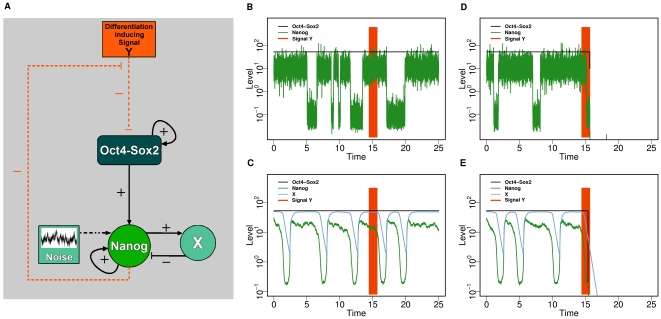
Simulated system dynamics in response to differentiation signals. (**A**) Hypothetical regulatory effect of the double-negative feedback from Nanog on Oct4-Sox2 to prevent the transmission of the differentiation inducing signal Y (dashed red lines). (**B–E**) Typical time courses of Oct4-Sox2 (black), Nanog (green), and X (blue) concentrations in response to a transient differentiation signal (applied within the red shaded regions) are provided for the fluctuation (B,D) and the oscillation scenario (C,E). The differentiation signals lead to a down-regulation of Oct4-Sox2 level only when they hit a cell in the Nanog-low state (D,E). As a consequence of the proposed interaction network this down-regulation irreversibly disables the variability of Nanog levels in both the fluctuation and the oscillation scenario.

Concluding from these observations we propose a novel interpretation for the regulating function of Nanog in maintaining the pluripotent ES cell state. In our explanation the Nanog levels critically regulate the transmission of differentiation inducing signals and thus control the propensity for differentiation. To study this concept in a quantitative fashion we have further amended the proposed network in Figure 1 by considering an indirect double-negative feedback loop from Nanog on the Oct4-Sox2 heterodimer which is mediated by an additional signal termed Y (Figure 6A). In simple words, the feedback loop is constructed such that a sufficiently high concentration of Nanog is able to inhibit the action of a differentiation inducing signal Y. In contrast, the differentiation inducing signal Y can exert a negative influence on the central pluripotency genes Oct4 and Sox2 in case of a low Nanog concentration. This double-negative feedback is a reinterpretation of the positive feedback which is generally assumed for Nanog regulating the expression of Oct4 and Sox2 [20], [35].

As demonstrated, there are at least two different mechanisms (i.e. the fluctuation and the oscillation scenario) to explain variations and reversible changes in the Nanog levels. Figure 6 (B–E) illustrates the systems response to differentiation inducing signals occurring at different time points (indicated by red bars) for both scenario. Whereas these signals are not able to induce a down-regulation of Oct4-Sox2 levels in case of high Nanog concentrations (Figure 6B and C) they do so if hitting an ES cell in the transient Nanog-low state (Figure 6D and E), independently of the scenario. These simulation results are consistent with experimental results about Nanog overexpression or knock-out [8] and support the view that differentiation and the final down-regulation of the central pluripotency genes Oct4 and Sox2 is only achieved if the (protective) Nanog concentrations are in the low-state. As both, the fluctuation and the oscillation scenario, are in principle able to quantitatively influence the fraction of ES cells being in either the Nanog-high or the Nanog-low state, they would both be suitable to control the efficiency of ES cell differentiation. Furthermore, our analysis suggests that differentiation induction is potentially regulated by a two-level mechanisms: (1) the regulation of the Nanog concentration (i.e., a *“gate-keeper” mechanism*), and (2) the absence or presence of differentiation inducing signals (i.e., an *induction event*).

In terms of the reprogramming of tissue cells into ES cell-like cells (generally referred to as induced pluripotent (iPS) cells), the role of Nanog is still controversial. On one hand Nanog is not necessary to reprogram somatic cells into a pre-iPS cell state, but critical to access the pluripotent ground state (i.e. a fully reprogrammed state) of ES cells [15], [16], [19]. Endogenous Nanog levels are only re-established in later phases of the reprogramming process [19]. Furthermore, it appears that the artificial induction of constantly high Nanog levels is sufficient to almost completely restrict the ES cell population from undergoing differentiation [11]. This whole spectrum of observations can consistently be explain by the interaction network proposed in Figure 6A in which Nanog is not causal for the establishment of pluripotency although it acts as a central control element for the maintenance of the pluripotent state.

## Discussion

Previous network models of the central pluripotency genes regularly assumed a direct positive feedback regulation from Nanog acting on Oct4 and Sox2. We have stated above that such a network configuration is not able to explain the experimentally observed Nanog fluctuations in the presence of constantly high Oct4/Sox2 levels. Therefore, we propose a simplified core network (Figure 1) in which the direct feedback loop from Nanog to Oct4/Sox2 is initially neglected. However, illustrating the role of Nanog as a central “gate-keeper” for the regulation of pluripotency we reinterpret this interaction between Nanog and Oct4/Sox2 as a double negative feedback that controls the penetration of differentiation inducing signals.

Starting from the simple core network that is able to show bistability (outlined in Figure 1, frame (A)) our mathematical model analysis demonstrates the existence of several, qualitatively different mechanisms that are able to explain the experimentally observed variability of Nanog expression in ES cells. Although the two presented concepts (i.e. fluctuation/oscillation scenario) consistently and equally well explain the currently available experimental data, they imply fundamentally different regulatory principles.

In the *fluctuation scenario*, changes between the Nanog-high and the Nanog-low state are induced by internal system perturbations. One possible assumption is that these perturbations result from a stochastic variability in the gene expression levels. In this sense, stochasticity takes a *functional* role in this scenario, i.e. it is the causal reason for the observed alternation between the two Nanog states. The frequency of state alterations can be controlled by the degree of the stochastic fluctuations (small variance – no/few state changes, large variance - frequent state changes), wherefore it can be regarded as an explicit control parameter. Such a “noise-driven” regulation has been proposed in the context of other systems, too [27], [29], [30].

In contrast, state transitions between the Nanog-high and the Nanog-low state in the *oscillation scenario* are explained as a consequence of deterministic system behaviour, namely a stable limit cycle. Although the deterministic oscillation behaviour is assumed to be overlaid by stochastic background fluctuations in the expression levels of Nanog, these have no regulatory consequence in this scenario. However, they are necessary to account for the experimentally observed variability of Nanog expression even within the Nanog-low and the Nanog-high state.

Alongside these potentially relevant TF interactions that determine the Nanog concentration in the very first place, the phenomenological appearance of a heterogeneous cell population is also clearly influenced by the processes of cell division and differentiation. For example, a higher proliferation rate of the Nanog-high cells in contrast to the Nanog-low cells would further skew the bimodal distribution. Such a mechanism could explain the reconstitution of the Nanog-high population (from a selection of Nanog-low cells) even with a much smaller transition rate for Nanog-low to Nanog-high than estimated in our model. However, as the proliferation effect can always be compensated by adjusting the particular transition rates, we argue that this effect does only quantitatively but not qualitatively alter our conclusions. Furthermore, beside those cells that have retained the ability to revert back into the Nanog-high state, the population of Nanog-low cells might also contain cells that have already initiated further differentiation including the irreversible down-regulation of Nanog and other relevant pluripotency genes. Purely based on their Nanog expression level these cells are most likely inseparable (as complete Nanog down-regulation and low level expression of Nanog can be expected to read out similarly in the assay) although they behave differently in response to the self-renewal conditions.

Our model explanations are not exclusive and there are alternative ways to explain the phenomena of varying Nanog levels. It is, for example, always possible to extend the proposed core network by additional regulators. Thus, it should be emphasized that the regulatory pathways, i.e. the edges in the proposed network graph, are not necessarily direct interactions. They are a minimal representation of the regulatory principles, potentially representing overall effects of more complex, indirect regulatory loops. Furthermore, the existence of time delays in the regulation network – a mechanism that has not been considered in our current analysis – is a possibility to explain Nanog oscillations.

Another particular mechanism, generating reversible Nanog variations, has recently been proposed by Kalmar and co-workers [35]. These authors suggest that neither bistability nor an oscillatory attractor (such as a limit cycle) is necessary to explain the varying Nanog levels. As an alternative they propose an excitable system that produces noise-induced transient “excursions” from a single steady state at high Nanog levels. Although this is an possible explanation on the basis of a system with simple dynamics (i.e. uni-stability), it is questionable whether or not the time scale of the transient escapes from the steady state are sufficiently long to explain the sojourn of cells in the Nanog-low state. Interestingly, Kalmar et al. also propose a potentially negative feedback effect on Nanog in case of high Oct4 levels. The precise mechanism (direct transcriptional repression or involving co-factors) and the regulation of this feedback are of similar interest as the proposed regulations of Nanog in the fluctuation and oscillation scenarios suggested by us. The simultaneous and continuous monitoring of Nanog and Oct4 expressions on the single cell level is an appropriate experimental strategy to address these regulations.

A distinction of the two proposed scenarios of Nanog regulation – fluctuation vs. oscillation – has important implications for further investigations, in particular on the selection of the targets for differentiation control. Whereas experimental evidence for the fluctuation scenario would focus on regulatory mechanisms that are able to modulate the degree of noise in terms of increasing or decreasing the variance σ^2^, opposing evidence for the oscillation model endorses the search for a structural cofactor, such as a negative regulator for Nanog. Besides other transcription factors, such as Gata6 or p53, that are know to repress Nanog expression [32], [38], also signalling pathways such as Notch signalling [39] and caspase-3 [40] or regulatory structures such as the NODE (Nanog and Oct4 associated deacetylase) complex [34] are potential candidates for the proposed co-factor X.

In particular, the fibroblast growth factor 4 (FGF4), which is a target gene of Oct4/Sox2 and which acts as an auto-inductive stimulus for ES cells to exit the self-renewal program through the activation of the Erk pathway [41], has already been discussed in this context. The FGF4/Erk signalling does not specify lineage commitment but it enables further inductive signals to act on the pluripotency network and has been suggested to control the transition of cultured ES cells between the two states, Nanog-high and Nanog-low [16]. Therefore, the FGF4/Erk pathway might be a regulator mechanism of the proposed “gate-keeper” function of Nanog for the differentiation of ES cells. Moreover, there is evidence that the Erk pathway induces Nanog repression [42], but there is so far no indication for an activation of FGF4 by Nanog itself.

**Figure 7:**
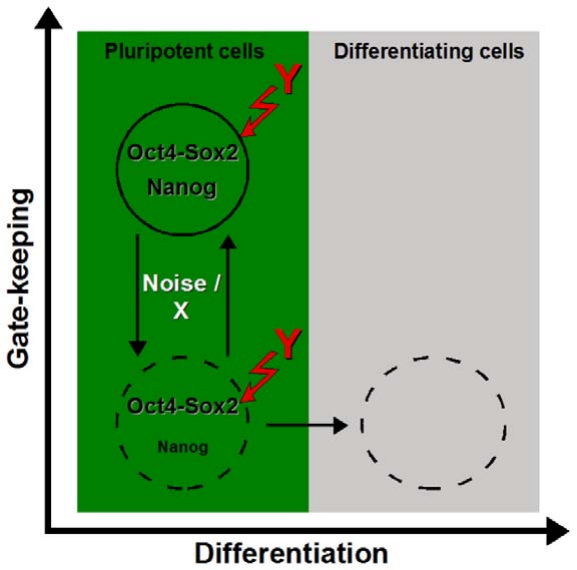
Two-level process for the induction of differentiation. Pluripotent ES cells can reversibly change between the Nanog-low and Nanog-high subpopulation, controlled by either of the two proposed regulatory mechanisms (indicated on the left side, vertical dimension). However, in the horizontal dimension the Nanog-low cells are more prone to differentiation inducing signals compared to the Nanog-high cells. As a consequence, some of the Nanog-low cells lose the ability to change back into the Nanog-high state by further down-regulation of Nanog and other pluripotency genes (horizontal dimension). In this visualisation the spatial dimensions correspond to the two-level mechanism: gate-keeping facilitated by variable Nanog concentrations – vertical dimension, induction of differentiation – horizontal dimension.

All the above observations are consistently summarized in a two-step concept to explain the induction of differentiation. In the first place, Nanog concentrations within individual cells are subject to reversible changes. We have proposed two possible mechanisms how such fluctuations can possibly be explained in the context of a bistable network configuration in which Nanog levels alternate between a high and a low-state. Depending on the parameter configurations the fraction of cells in either of the two states can be influenced. As discussed above, the activation or inactivation of the FGF4/Erk pathway is a potential candidate mechanism for this regulation of the Nanog-high/low transition either by affecting the negative Nanog/X feedback loop (in the oscillation scenario) or by regulating the degree of the Nanog fluctuations (in the fluctuation scenario). Although the Oct4/Sox2-high and Nanog-high state is considered to be the true ground state of ES cells [13], the transient state expressing high Oct4/Sox2 levels but low Nanog levels still retains the potency for ES cell maintenance and pluripotency [11], [19]. However, these transient cells are generally more prone to undergo further differentiation in response to differentiation inducing signals as compared to the Oct4/Sox2-high and Nanog-high cells. In this respect, the reversible down-regulation of Nanog is the first step of the differentiation sequence (*“gate-keeper” mechanism*) while the actual phenotypic differentiation is initiated only in the presence of appropriate signals (*induction event*). The nature of this differentiation inducing signals is still speculative as both internal and external effects could potentially propagate such action. However, our model provides a mathematical framework in which the concept of a two level mechanism (Figure 7) for the induction of differentiation is consistently embedded.

Summarizing, our modelling approach shows that transient variations of Nanog levels can be explained by rather simple interaction networks. Integrating these interaction networks in many individual cells the observed bimodal distribution of the cellular Nanog concentration is reconstructed. Based on our results we argue that the transient down-regulation of the Nanog expression in ES cells is a functional feature of these populations to regulate the susceptibility for differentiation signals. The particular culture conditions ultimately influence the fraction of ES cells in the Nanog-high vs. Nanog-low state and, consequently, their propensity for differentiation. Changes in the culture condition might adjust transition rates between the Nanog-high and -low state without ultimately shifting the whole population in one or the other state. In this respect, our suggested molecular interaction networks qualify as potential candidates to translate the observed population heterogeneity onto a molecular basis. Our theoretical results clearly support the concept that the temporary down-regulation of Nanog might act as a “gate-keeper” for the initiation of differentiation and the final shutting down of further genes related to pluripotency such as Oct4 and Sox2, as suggested by [12] and [13]. Although the proposed model is just a first step we argue that the quantitative understanding of the governing principles of the pluripotency network, which requires the application of mathematical approaches, is an essential key to a comprehensive perception of ES cell behaviour. Furthermore, this understanding is crucial to interfere and to control the fate of (stem) cells in the context of targeted differentiation or reprogramming settings.
